# The p53 tumor suppressor regulates AKR1B1 expression, a metastasis-promoting gene in breast cancer

**DOI:** 10.3389/fmolb.2023.1145279

**Published:** 2023-09-14

**Authors:** Carolina Di Benedetto, Carla Borini Etichetti, Nabila Cocordano, Alejo Cantoia, Evelyn Arel Zalazar, Silvio Bicciato, Mauricio Menacho-Márquez, Germán Leandro Rosano, Javier Girardini

**Affiliations:** ^1^ Department of Radiation Oncology, University of California San Francisco, San Francisco, CA, United States; ^2^ Instituto de Fisiología Experimental de Rosario (IFISE), Consejo Nacional de Investigaciones Científicas y Técnicas (CONICET), Universidad Nacional de Rosario, Rosario, Argentina; ^3^ Instituto de Inmunología Clínica y Experimental de Rosario (IDICER), Consejo Nacional de Investigaciones Científicas y Técnicas (CONICET), Universidad Nacional de Rosario, Rosario, Argentina; ^4^ Unidad de Espectrometría de Masa, Instituto de Biología Molecular y Celular de Rosario (IBR), Consejo Nacional de Investigaciones Científicas y Técnicas (CONICET), Universidad Nacional de Rosario, Rosario, Argentina; ^5^ Department of Life Sciences, University of Modena and Reggio Emilia, Modena, Italy

**Keywords:** aldo–keto reductase 1 B1, p53, metastasis, breast cancer, aldo–keto reductases, electron transport chain, mitochondria, proteomics

## Abstract

Alteration of metabolism in cancer cells is a central aspect of the mechanisms that sustain aggressive traits. Aldo–keto reductase 1 B1 (AKR1B1) catalyzes the reduction of several aldehydes to alcohols consuming NADPH. Nevertheless, the ability of AKR1B1 to reduce different substrates renders difficult to comprehensively ascertain its biological role. Recent evidence has implicated AKR1B1 in cancer; however, the mechanisms underlying its pro-oncogenic function remain largely unknown. In this work, we report that AKR1B1 expression is controlled by the p53 tumor suppressor. We found that breast cancer patients bearing wild-type *TP53* have reduced *AKR1B1* expression. In cancer cell lines, p53 reduced AKR1B1 mRNA and protein levels and repressed promoter activity in luciferase assays. Furthermore, chromatin immunoprecipitation assays indicated that p53 is recruited to the AKR1B1 promoter. We also observed that AKR1B1 overexpression promoted metastasis in the 4T1 orthotopic model of triple-negative breast cancer. Proteomic analysis of 4T1 cells overexpressing AKR1B1 showed that AKR1B1 exerts a marked effect on proteins related to metabolism, with a particular impact on mitochondrial function. This work provides novel insights on the link between the p53 pathway and metabolism in cancer cells and contributes to characterizing the alterations associated to the pathologic role of AKR1B1.

## 1 Introduction

Aldo–keto reductase 1 B1 (AKR1B1), a cytosolic enzyme belonging to the aldo–keto reductase superfamily, catalyzes the reduction of several aldehydes to alcohols consuming NADPH (Pastel et al., 2012). Glucose reduction by AKR1B1 has attracted attention because of its consequences in diabetes (Tang et al., 2012). This is the first step of the polyol pathway, which consists of two reactions that transform glucose into fructose. The first reaction is the reduction of glucose to sorbitol catalyzed by AKR1B1, the rate-limiting step of the pathway (Kinoshita, 1990). The pathway is completed by the oxidation of sorbitol to fructose, catalyzed by sorbitol dehydrogenase, using NAD^+^ as a cofactor. Fructose may be later phosphorylated to fructose-6-P and reincorporated into glycolysis or used to generate advanced glycation products (Srivastava et al., 2005). Recent experimental evidence has shown that AKR1B1 may exert pro-oncogenic effects. For instance, in breast cancer cell lines, AKR1B1 was shown to cooperate with migration and invasion *in vitro* (Wu et al., 2017b). Likewise, AKR1B1 knockdown reduced tumor formation *in vivo* and lung colonization upon vein tail injection in immunocompromised mice. In addition, levels of cancer stem cell markers were reduced upon AKR1B1 silencing in lung and breast cancer cell lines (Schwab et al., 2018). To date, a comprehensive understanding of the biological role of AKR1B1 remains elusive, mostly due to its ability to reduce other substrates. These include aldoses such as xylose and glyceraldehyde, as well as methylglyoxal, prostaglandin H2 (PGH2), and lipid peroxides, among others (Bresson et al., 2012; Tammali et al., 2012). Therefore, the available evidence suggests that alteration of different aspects of cell metabolism underlies the pathologic effects of AKR1B1.

Alteration of metabolism is a hallmark of cancer cells (Hanahan and Weinberg, 2011). Following the initial observations showing a switch to aerobic glycolysis and lactate production, intense research in the last years has brought a more complex scenario to light. Cancer cells often show enhanced nutrient uptake and extensive reshaping of intermediate metabolite usage among anabolic and catabolic pathways (Pavlova and Thompson, 2016). A growing body of evidence has documented the role of the tumor suppressor p53 in the regulation of cell metabolism. p53 constitutes the central hub of a complex signaling pathway activated in response to different stress signals (Kastenhuber and Lowe, 2017). Upon stabilization and activation, p53 can regulate the transcription of specific target genes depending on the stimulus and context.

The p53 pathway can control different aspects of glucose metabolism. For example, p53 represses the expression of glucose transporters (Schwartzenberg-Bar-Yoseph et al., 2004) and negatively regulates glycolysis through its transcriptional targets TIGAR (Bensaad et al., 2006), mir-34a (Napoli and Flores, 2017), and Parkin (Zhang et al., 2011). p53 also enhances the tricarboxylic acid (TCA) cycle by repressing lactate release on the extracellular medium and inducing GSL2 expression (Napoli and Flores, 2017). The emerging picture suggests that p53 contributes to maintaining moderate levels of glucose uptake and glycolytic flux, in concert with tightly regulated oxidative phosphorylation. In addition, p53 inhibits the activation of SREBP1/2 transcription factors, leading to different effects on lipid metabolism, including the downregulation of genes of the mevalonate pathway (Moon et al., 2019; Borini Etichetti et al., 2020).

Considering that p53 exerts a concerted regulation of different aspects of cell metabolism, we hypothesized that it might also affect enzymes involved in the polyol pathway. Indeed, our work shows that AKR1B1 expression is repressed at the transcriptional level by p53 in cancer cell lines and that AKR1B1 mRNA levels are reduced in breast cancer patients’ tumors retaining wild-type *TP53* (the gene encoding p53), as compared to tumors bearing mutations in this gene. Using an immunocompetent mouse model of breast cancer, we also found that AKR1B1 overexpression accelerates tumor growth and promotes metastasis. Thus, our results indicate that loss of the wild-type (wt) p53 repressive effect on AKR1B1 expression could collaborate with breast cancer aggressiveness. In addition, proteomic analyses performed in breast cancer cells to explore the mechanisms underlying the AKR1B1 pro-oncogenic role indicate that AKR1B1 overexpression highly impacts cell metabolism.

## 2 Results

**FIGURE 1:**
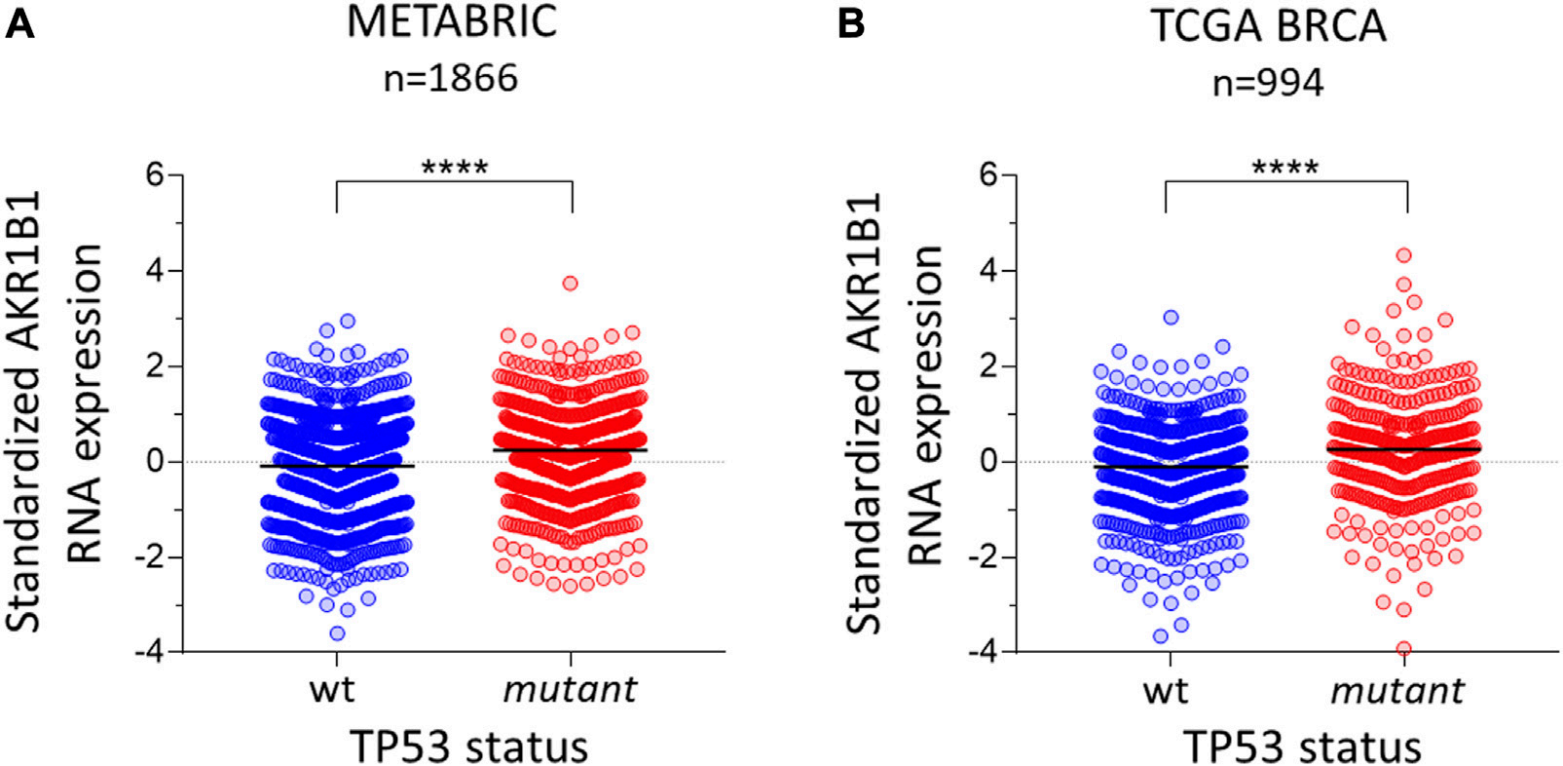
*AKR1B1* RNA expression is linked to *TP53* status in breast cancer. Standardized RNA levels of *AKR1B1* RNA are lower in wild-type *TP53* tumors than in mutant *TP53* tumors in breast cancer samples from **(A)** METABRIC (n = 1866) and **(B)** TCGA (n = 994). The two-tailed Mann–Whitney test was performed; ****p < 0.0001.

**FIGURE 2:**
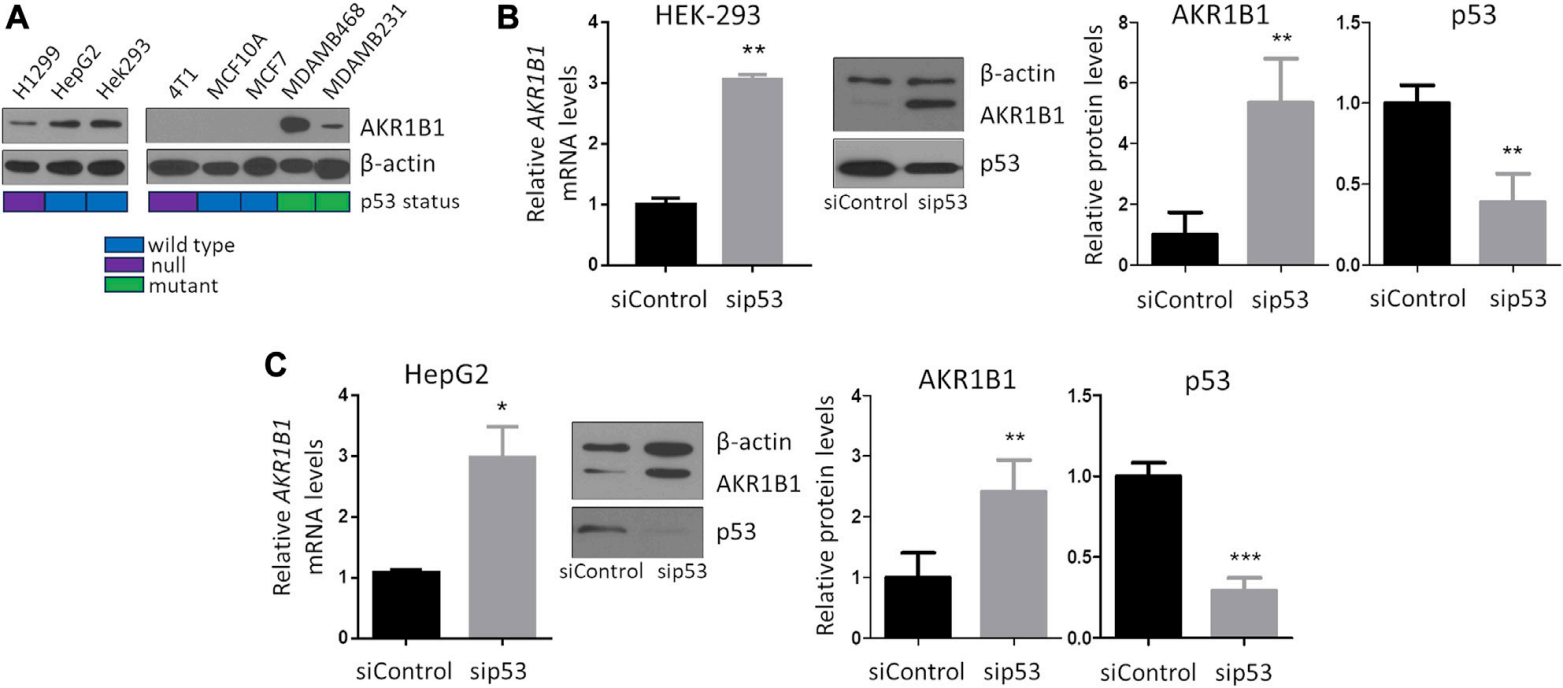
p53 represses AKR1B1 expression. **(A)** AKR1B1 protein levels are highly variable in a panel of human and murine cell lines, as observed by Western blot analysis. Colored boxes represent TP53 status, obtained from the Cancer Cell Line Encyclopedia or from the literature (Yerlikaya and Erin, 2008). **(B)** Silencing of p53 increases AKR1B1 expression in **(B)** HEK-293 and **(C)** HepG2 cells. Cells were transfected with siRNA-targeting p53 or control (sip53 or siControl), and AKR1B1 expression levels were determined by qPCR or Western blot. p53 silencing was confirmed by Western blot. Intensity of Western blot bands was quantified using ImageJ and normalized to β-actin levels. Two-tailed *t*-test, *p < 0.05, **p < 0.01, and ***p < 0.001; n = 4.

To investigate whether the p53 pathway affects *AKR1B1* expression, we first analyzed public cancer patients’ databases looking for correlations between p53 status and *AKR1B1* mRNA levels. We focused on breast cancer patients from METABRIC and TCGA databases, both of which contain information on the mutational status of the *TP53* gene. We found that patients retaining wt *TP53* alleles showed a significant reduction in *AKR1B1* mRNA levels compared with patients with mutations in this gene (Figures 1A,B). These results indicate that inactivation of *TP53* is correlated with enhanced *AKR1B1* mRNA levels in breast cancer patients and suggested us that p53 negatively regulates *AKR1B1* expression. To further explore this hypothesis, we wondered whether p53 may affect *AKR1B1* expression in cancer cell lines. First, we performed Western blot analysis in a panel including human and murine cell lines (Figure 2A). Interestingly, we observed striking differences in the expression levels of this enzyme, which was present in some cell lines but not detected in others. In line with this observation, AKR1B1 mRNA expression levels obtained from RNA sequencing data publicly available on the Cancer Cell Line Encyclopedia showed a similar expression pattern (Supplementary Figure S1A). In addition, AKR1B1 mRNA and protein expression levels are highly correlated in breast cancer cell lines (Supplementary Figure S1B), suggesting that AKR1B1 protein levels are mainly regulated at the transcriptional level. It should be noted that, considering the human breast cell lines on Figure 2A, AKR1B1 protein expression seems to correlate with p53 status, since the two cell lines that express high AKR1B1 protein levels (MDAMB231 and MDAMB468) have mutations in *TP53*, while AKR1B1 protein expression was not detected in wt p53 cells (MCF10A and MCF7).

To investigate whether p53 represses AKR1B1 expression, we concentrated on HEK-293 and HepG2 cell lines, in which p53 is not mutated and AKR1B1 was detectable by Western blot. We found that *AKR1B1* mRNA levels increased upon silencing of endogenous p53, and a similar effect was observed on AKR1B1 protein levels (Figures 2B,C), suggesting that wt p53 represses *AKR1B1* expression at the transcriptional level in these cancer cell lines.

**FIGURE 3:**
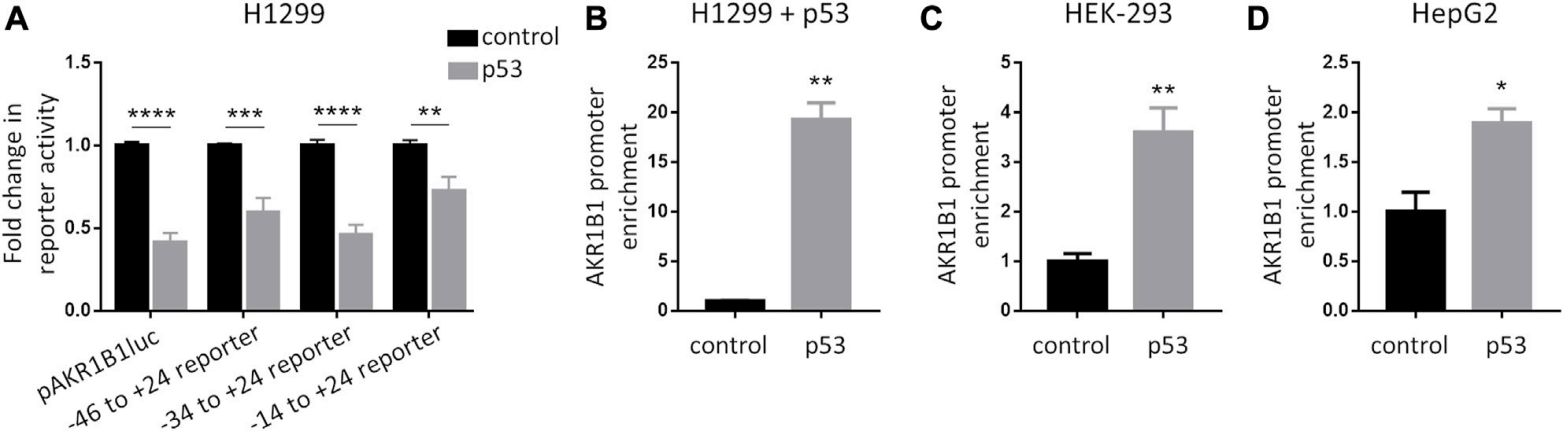
p53 is recruited to the AKR1B1 promoter. **(A)** p53 reduces AKR1B1 promoter activity. Luciferase assays were performed in H1299 cells co-transfected with the indicated reporter plasmid for the AKR1B1 promoter deletions (pAKR1B1luc; pAKR1B1luc100; pAKR1B1luc70; or pAKR1B1luc40), a plasmid expressing p53 (pCDNA3-p53) or empty vector (pCDNA3) as control, and pCMV-β-gal. Values were normalized to β-galactosidase activity and expressed as fold change relative to cells transfected with empty vector. Two-way ANOVA test, **p < 0.01, ***p < 0.001, and ****p < 0.0001; n = 4 for pAKR1B1luc; and n = 3 for pAKR1B1luc100, pAKR1B1luc70, and pAKR1B1luc40. **(B)** p53 is recruited to the AKR1B1 promoter in H1299 (p53 null) cells transfected with pCDNA3-p53 or pCDNA3 as control. ChIP immunoprecipitation was performed using the anti-p53 antibody (DO1). **(C)** Endogenous p53 is recruited to the AKR1B1 promoter in HEK-293 cells. ChIP immunoprecipitation was performed using the anti-p53 antibody (DO1) or a control antibody. **(D)** Endogenous p53 is recruited to the AKR1B1 promoter in HepG2 cells. ChIP immunoprecipitation was performed using the anti-p53 antibody (DO1) or a control antibody. A two-tailed *t*-test was performed, *p < 0.05 and **p < 0.01.

To further characterize the role of p53 on *AKR1B1* expression, we analyzed its effect on promoter activity. We generated the reporter pAKR1B1luc by cloning a fragment of the *AKR1B1* promoter, including the region −1785 to +24 from the transcription start site, into the pGL3 vector. Upon co-transfection with pAKR1B1luc in H1299 cells (p53 null), we found that p53 inhibited promoter activity in luciferase assays (Figure 3A). To identify sequences involved in the repressive effect of p53 on the *AKR1B1* promoter, we generated a series of reporters containing deletions at the 5′end of the promoter, covering the region from −1785 to −14 (Supplementary Figure S2A). When we tested these reporters in luciferase assays, we found that p53 repressed *AKR1B1* promoter activity in all cases (Figure 3A), indicating that DNA regions that sustain the observed effect of p53 are located close to the transcription start site on the *AKR1B1* promoter.

Next, we performed chromatin immunoprecipitation (ChIP) assays to analyze if p53 is recruited to the *AKR1B1* promoter. Upon expression of p53 in H1299 cells and immunoprecipitation with an anti-p53 antibody, we found a significant enrichment of the region between −46 and +24 of the *AKR1B1* promoter in the immunoprecipitated DNA, compared to H1299 control cells transfected with an empty vector and, therefore, not expressing p53 (Figure 3B). We performed similar experiments but immunoprecipitating endogenous p53 in HEK-293 and HepG2 cell lines. Here, a significant enrichment of the *AKR1B1* promoter fragment was found relative to the control condition, in which DNA was immunoprecipitated with an unrelated antibody (Figures 3C,D). Moreover, we also found an *AKR1B1* promoter enrichment when we performed ChIP experiments upon p53 protein stabilization upon doxorubicin treatment in HCT116 cells (Supplementary Figure S2B). Taken together, our data show that p53 represses *AKR1B1* transcription and that it is recruited to the *AKR1B1* promoter. In silico analysis of the *AKR1B1* promoter sequence failed to identify a p53 responsive element, suggesting that p53 is recruited by an indirect mechanism, probably involving the interaction with other proteins present on the chromatin.

**FIGURE 4:**
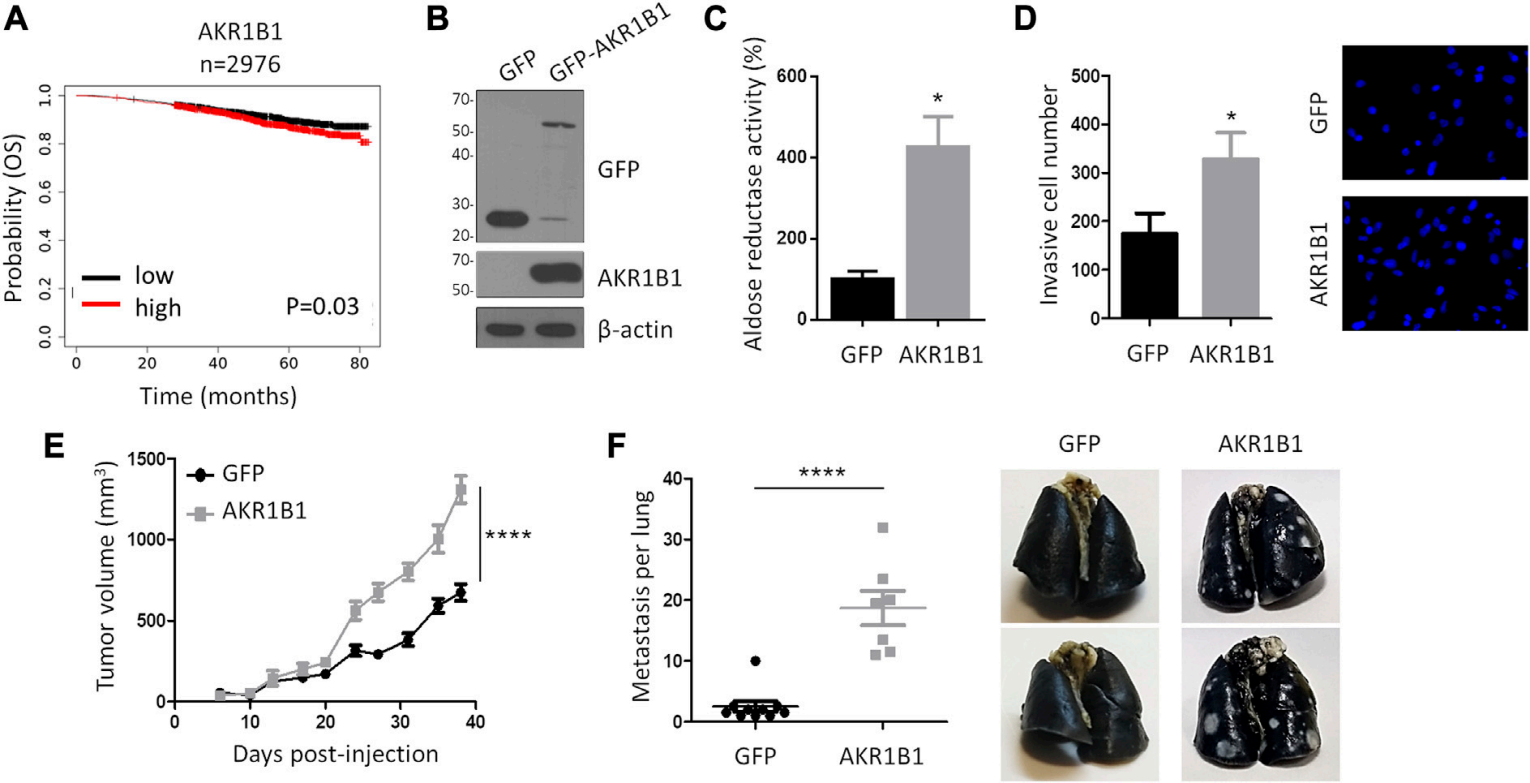
AKR1B1 promotes invasion *in vitro* and metastasis *in vivo*. **(A)** Kaplan–Meier plot showing breast cancer patients’ overall survival probability over time based on AKR1B1 mRNA expression. The analysis was performed using RNA-Seq data available on KM plotter for 2,976 breast cancer patients. Red and black curves represent samples that show high and low gene expression levels, respectively, relative to the median AKR1B1 expression value. High levels of AKR1B1 RNA are significantly correlated with breast cancer patients' poor prognosis. A log-rank test was performed. **(B)** Western blot analysis confirms AKR1B1-GFP overexpression in 4T1 cells. **(C)** AKR1B1 overexpression in 4T1 cells increases aldose reductase activity. Enzymatic activity was quantified in cell extracts, using D + -xylose as the substrate and following absorbance at 340 nm, corresponding to NADPH oxidation. A two-tailed *t*-test was performed, *p < 0.05. **(D)** AKR1B1 overexpression in 4T1 cells increases invasion *in vitro*. 4T1-AKR1B1 (AKR1B1) or 4T1-GFP (GFP) cells were plated on the upper side of Matrigel-coated Transwell chambers. After 24 h, cells on the lower surface were stained and quantified. Representative images are shown. A two-tailed *t*-test was performed, *p < 0.05; n = 4. **(E)** 4T1-AKR1B1 or 4T1-GFP cells were injected in the fat pad of female BALBc mice, and tumor growth was monitored (4T1-AKR1B1 n = 7, 4T1-GFP n = 10). Non-linear regression for exponential growth fit was performed, ****p < 0.0001. **(F)** AKR1B1 overexpression increases metastatic potential of 4T1 cells to the lung. After sacrifice, lung metastases were analyzed by intra-tracheal injection of India ink. Pictures show representative lungs obtained from the two groups. A two-tailed *t*-test was performed, ****p < 0.0001.

Our results suggest that AKR1B1 levels are tightly regulated in the presence of active p53. In contrast, p53 inactivation may promote AKR1B1 overexpression. Therefore, we hypothesized that AKR1B1 overexpression may cooperate with tumor progression. Accordingly, when we analyzed public databases, we found that breast cancer patients with high *AK1B1* mRNA levels displayed a significant reduction in overall survival, with a survival probability of 0.807 and 0.872 for patients expressing high and low *AKR1B1* mRNA, respectively (n = 2976, Figure 4A). Nevertheless, the role of AKR1B1 overexpression on metastasis has not been studied. Therefore, we took advantage of an orthotopic model of breast cancer, consisting in the transplantation of 4T1 cells in the mammary fat pad of BALB/c mice. These cells are derived from a murine triple-negative breast adenocarcinoma and develop tumors *in situ*, which can metastasize to other tissues, primarily the lungs (Lelekakis et al., 1999). In contrast to direct injection of tumor cells in the vein tail, this approach has the advantage of providing a model for the complete metastatic process. Moreover, the transplantation of murine cells allows the use of immunocompetent mice, thus considering the role of the immune system in the analysis (Aslakson and Miller, 1992). 4T1 cells with stable overexpression of GFP-AKR1B1 (4T1-AKR1B1) or GFP as control (4T1-GFP) were generated, and the presence of the fusion protein was confirmed by Western blot (Figure 4B). We also observed an increase in aldose reductase activity in extracts from 4T1-AKR1B1 cells, corroborating the overexpression of a functional protein (Figure 4C).

Since the effect of AKR1B1 overexpression on cell invasion *in vitro* has not been analyzed previously, we first performed Transwell assays on Matrigel-coated filters. We found that 4T1-AKR1B1 cells showed a significant increase in invasive behavior (Figure 4D). Next, 4T1-AKR1B1 or AKR1B1-GFP cells were injected into the mammary fat pad of female BALB/c mice, and tumor development was monitored periodically. As seen in Figure 4E, tumors arising from 4T1-AKR1B1 cells showed a significant increase in the growth rate, and the final mean tumor volume approximately doubled the mean volume of the control condition (1309.4 mm^3^ *versus* 673.4 mm^3^, respectively). Of note, three mice in the AKR1B1-overexpressing group died before experiment completion, at days 6, 35, and 37 post-injection. We also analyzed the presence of macroscopic metastases in the lungs. We observed that all mice in our study developed lung metastasis. However, the overexpression of AKR1B1 resulted in a dramatic increase in metastasis development, as mice injected with 4T1-AKR1B1 showed 37.43 pulmonary metastases on average, whereas 4T1-GFP control mice showed an average of five pulmonary metastases (Figure 4F), i.e., 4T1-AKR1B1 tumors produced a >7-fold increase in the number of lung metastases compared to the control. As it can be observed in the representative images shown in Figure 4F, pulmonary metastases were also much bigger in the 4T1-AKR1B1 group than in the control mice. Likewise, three metastases were detected in the spleen and one in the liver in the group of mice injected with 4T1-AKR1B1 cells, but macroscopic metastases were not detected in these organs in the control group (results not shown). Together, these results show, for the first time, that AKR1B1 overexpression promotes metastasis in an orthotopic breast cancer model *in vivo*.

**FIGURE 5:**
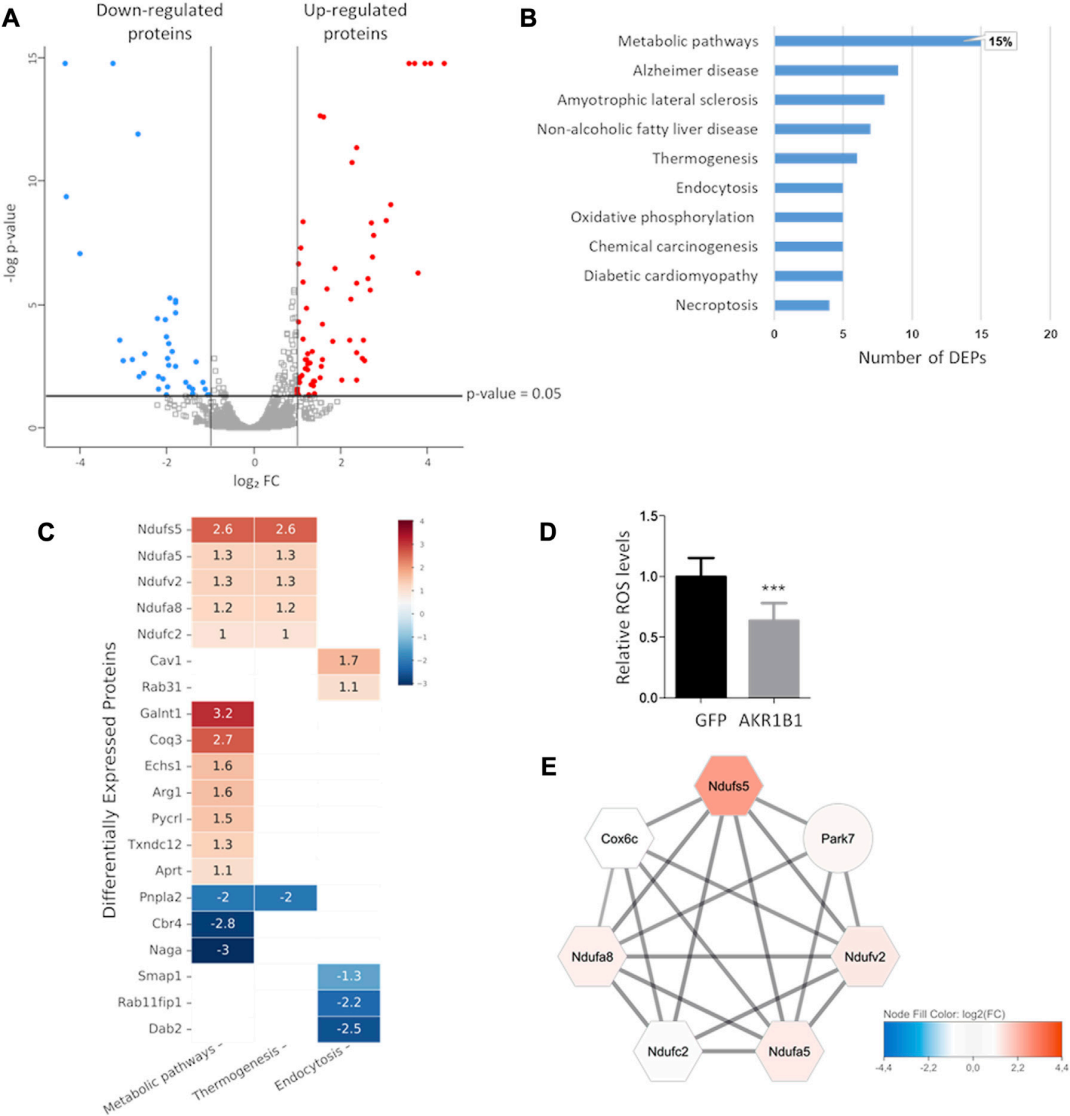
AKR1B1 overexpression highly impacts metabolism in 4T1 cells. **(A)** Volcano plot showing differential protein expression in 4T1-AKR1B1 cells relative to 4T1-GFP cells. Proteins identified by at least two unique peptides are represented (3822). Only proteins identified by at least two peptides, showing FC ≥ 2 (up- or downregulated) and *p*-value ≤ 0.05, were considered as differentially expressed proteins. Upregulated proteins are shown as red dots and downregulated as blue dots. The gray dots represent the proteins with unaltered expression. **(B)** Pathway enrichment analysis by the KEGG Mapper. Bar graph showing the KEGG categories enriched in DEPs from the proteomic analysis. The category “Metabolic pathways” included 15 DEPs, representing 15% of the DEPs identified. **(C)** Functional KEGG categories enriched in proteins affected by AKR1B1 overexpression. Heatmap showing selected functional categories defined using the KEGG mapper tool containing more than four DEPs. The color code indicates relative fold expression expressed as log2 FC, comparing 4T1-AKR1B1 with 4T1 GFP cells. The numbers correspond to the log2 FC of each DEP. **(D)** ROS levels decrease in 4T1-AK1B1 cells. 4T1-AKR1B1 or control cells were incubated with 5 µM 2′,7′-dichlorofluorescein diacetate for 45 min, and after washing, fluorescence was measured. Non-stained cell suspensions were used as blank. A two-tailed *t*-test was performed, ***p < 0.001. **(E)** A cluster related to the mitochondrial electron transport chain was defined by network analysis. The color code indicates relative fold expression expressed as log2 FC. Hexagons represent components of the mitochondrial electron transport chain. The solid black lines indicate functional and/or physical interaction defined by the software.

To gain more insight into the mechanisms underlying the oncogenic role of AKR1B1, we performed a global proteomic analysis. Protein extracts from 4T1-AKR1B1 and 4T1-GFP were analyzed following a label-free bottom–up mass spectrometry strategy. Based on our analysis, 3822 proteins were identified with at least two unique peptides and quantified. As evidenced in the volcano plot in Figure 5A, 99 proteins showed significantly altered abundances with a fold change (FC) of 2 or higher. Among them, 62 proteins were upregulated and 37 downregulated in 4T1-AKR1B1 cells compared with 4T1-GFP cells. Accordingly, we defined a list of differentially expressed proteins (DEPs), up- or downregulated, considering only proteins identified by two or more unique peptides with an FC of 2 or higher (*p*-value ≤ 0.05, Supplementary Table S1).

We performed a functional analysis of the identified DEPs to explore the effects of AKR1B1 overexpression on biological processes. Using the KEGG Mapper tool, we performed a pathway enrichment analysis. We found that the category metabolic pathways was the most enriched in the DEPs identified by our proteomic study. As shown in Figure 5B, this category includes 15 DEPs, compared with all the other categories that counted nine DEPs or less. These results reinforce the idea that AKR1B1 contributes to shape tumor cell metabolism. Among the upregulated proteins in the metabolic pathways category, we found several components of the mitochondrial electron transport chain (Figure 5C). For example, components of the ubiquinone oxidoreductase complexes Ndufs5, Ndufv2, Ndufa5, Ndufa8, Ndufc2, and Ndufaf2 were identified. Similarly, Coq3, a protein involved in coenzyme Q biosynthesis, was found upregulated. These results suggest that AKR1B1 overexpression may enhance oxidative phosphorylation. In agreement, previous reports showed that AKR1B1 downregulation reduced the oxygen consumption rate in the A549 cell line (Schwab et al., 2018). To further characterize the effect of AKR1B1 on oxidative stress, we measured reactive oxygen species (ROS) levels in 4T1-AKR1B1 cells. We found that AKR1B1 overexpression significantly reduced ROS levels compared to control cells (Figure 5D). AKR1B1 is known to protect cells against reactive compounds generated under oxidative stress (Shen et al., 2011; Sánchez-Gómez et al., 2016), suggesting that its detoxifying activities during the antioxidant response could counterbalance the consequences of increased oxidative phosphorylation.

We used the STRING protein interaction repository to outline the interaction networks between the identified DEPs that could help understand their functional relationships. In this case, we extended the DEP list considering proteins with log_2_FC ≥ 0.85 or log_2_FC ≤ −0.85 (*p*-value ≤ 0.05), in order to increase the number of interactors and, therefore, to highlight connections that may be unappreciated with a more restrictive criterion (Supplementary Table S1). The interactions retrieved from STRING were loaded onto the Cytoscape platform to perform network analysis, visualization, and clustering. As shown in Figure 5E, one highly interconnected cluster emerged from the network obtained (Supplementary Figure S3). This cluster contains upregulated proteins related to mitochondrial function, including the components of the ubiquinone oxidoreductase complex also identified by pathway enrichment analysis in KEGG, as well as Cox6c and Park7. Cox6c is a subunit of cytochrome c oxidase, another key component of the electron transport chain. Although Park7 is not included in the same functional category by the KEGG mapper, it is related to mitochondria. Park7 loss is associated to mitochondria fragmentation and reduced membrane potential (Wang et al., 2016). Interestingly, this protein has been shown to promote oncogenic mechanisms in breast cancer (Kim et al., 2021).

Other DEPs present in the KEGG metabolic pathways category on Figure 5C are also related to different aspects of metabolism, such as protein glycosylation and amino acid and nucleotide biosynthesis. Nevertheless, a clear functional interaction among them is not so evident, and a strong network could not be clearly defined in our network analysis. In addition, some DEPs related to lipid metabolism were also present in the metabolic pathways category. For example, Echs1 was upregulated upon AKR1B1 overexpression. This enzyme catalyzes the hydration of short-chain enoyl-CoAs, as part of the process of fatty acid β-oxidation in mitochondria (Hu et al., 2022). Among downregulated proteins, we found Cbr4, a mitochondrial carbonyl reductase involved in mitochondrial fatty acid synthesis (FAS) (Venkatesan et al., 2014). We also found Pnpla2 as downregulated. Pnpla2 catalyzes the first step of lipolysis, generating diacylglycerols from triglycerides. Although its role in cancer is not fully understood, some evidence indicates a cooperation with tumor suppression mechanisms. For example, in humans, *PNPLA2* deletion was found in sarcoma and liposarcoma, and a Pnpla2/Hsl double-knockout mice model developed liposarcoma (Wu et al., 2017a). Taken together, our proteomic analyses show that AKR1B1 overexpression deeply alters cell metabolism.

## 3 Discussion

The acquisition of aggressive traits in cancer cells is often associated to extensive reshaping of metabolic circuits. Such alterations may create a dependence on specific metabolic programs, thus providing potential avenues for therapeutic intervention. However, in order to successfully interfere with cancer-specific metabolic features, a more profound knowledge of the mechanisms that regulate cancer cell metabolism is required.

In this work, we showed that aldo–keto reductase AKR1B1 is a strong promoter of metastasis *in vivo*, and its upregulation exerts a marked effect on proteins related to metabolism in breast cancer cells. We also showed that p53 represses AKR1B1 expression by inhibiting its promoter activity. The analysis of breast cancer patients’ databases showed a significant decrease in *AKR1B1* RNA expression in tumors retaining wt *TP53*. Based on our results, we hypothesize that p53 contributes to tightly regulating *AKR1B1* transcript levels in physiological conditions. During tumor progression, blockade of p53 function may contribute to increase *AKR1B1* RNA levels. Mutations in *TP53* are among the most frequent alterations in human cancer. In breast cancer, *TP53* mutation is found in approximately 30% of cases, and it is correlated with poor clinical outcome (Silwal-Pandit et al., 2014). In cases maintaining wt alleles, p53 function is often inhibited by the aberrant activity of regulators (Hainaut and Pfeifer, 2016). AKR1B1 overexpression in cancer may also be a consequence of additional p53-independent mechanisms. For instance, AKR1B1 transcription is enhanced by Twist2, a well characterized promoter of epithelial–mesenchymal transition that cooperates with tumor aggressiveness (Wu et al., 2017b). Our work provides the first demonstration that AKR1B1 overexpression enhances metastasis from primary tumors, in a model of breast cancer in immunocompetent mice. These results provide strong support to the notion that an increase on AKR1B1 levels during tumor progression may constitute a critical event to develop an aggressive phenotype.

The analysis of the effect of AKR1B1 overexpression on the proteome demonstrated a marked impact on proteins related to metabolism. Our results suggest that the enhancement of the mitochondrial electron transport chain could be linked to AKR1B1 oncogenic function. Moreover, we found that AKR1B1 overexpression reduced ROS levels in 4T1 cells. Although clearly involved in tumorigenesis and tumor progression, accumulating evidence has made it clear that the role of ROS in cancer cell behavior is extremely complex and context dependent (Cheung and Vousden, 2022). It can be hypothesized that reduction of ROS levels by AKR1B1 could protect cells from excessive damage under conditions of enhanced electron transport chain activity, as suggested by our proteomic studies.

We also observed an alteration of mitochondrial enzymes related to fatty acid metabolism that suggests an enhancement of fatty acid β-oxidation. The alteration of lipid metabolism in cancer cells often includes enhanced fatty acid β-oxidation as a source of NADH, FADH_2_, and NADPH (Hu et al., 2022), while relying in cytosolic FAS for lipid generation (Röhrig and Schulze, 2016). In this context, Cbr4 downregulation may reduce acetyl-CoA consumption for mitochondrial FAS, favoring its bioavailability for other reactions. Collectively, these results suggest that the alteration of mitochondrial function is a central feature of AKR1B1 pro-oncogenic function.

In summary, our work identifies AKR1B1 overexpression as an alteration related to loss of p53 function that promotes the acquisition of aggressive tumor phenotypes. Our proteomic analysis contributes to understanding the effects associated to AKR1B1 pathologic function, showing a complex scenario where AKR1B1 affects different aspects of cell physiology with a marked impact on metabolism.

## 4 Materials and methods

### 4.1 Analyses of cancer datasets

AKR1B1 mRNA expression (as z-scores relative to all samples) and *TP53* status data from The Cancer Genome Atlas (TCGA) breast cancer dataset (Koboldt et al., 2012) or the Molecular Taxonomy of Breast Cancer International Consortium (METABRIC) study (Curtis et al., 2012) were downloaded from cBioPortal (Cerami et al., 2012). AKR1B1 RNA expression levels in wt and mutant *TP53* tumors were compared using the Mann–Whitney test. Kaplan–Meier (KM) plots for AKR1B1 RNA expression in breast cancer were performed using KM plotter (https://kmplot.com/analysis/) (Győrffy, 2021). Patients were divided into two groups based on the median AKR1B1 expression value, and KM curves were compared using the log-rank (Mantel–Cox) test. AKR1B1 mRNA expression (as reads per kilobase per million mapped reads, RPKM), AKR1B1 protein levels, and TP53 status data in cancer cell lines, publicly available on the Cancer Cell Line Encyclopedia (Ghandi et al., 2019; Nusinow et al., 2020), were downloaded from cBioPortal.

### 4.2 Cell culture and drugs

HEK-293, HCT116, HEK293-GP, MDAMB231, MDAMB468, and MCF7 were cultured in DMEM medium (Gibco) supplemented with 10% fetal bovine serum (FBS, Gibco). MDAMB468 and MCF7 were also supplemented with 2 mM glutamine (Gibco). H1299 and 4T1 cells were cultured in RPMI 1640 medium (Gibco) supplemented with 10% FBS (Gibco). MCF10A cells were cultured in DMEM:F12 medium (Gibco) supplemented with 10% FBS (Gibco), 0.5 μg/mL hydrocortisone, 10 μg/mL insulin, 20 ng/mL epidermal growth factor, 100 ng/mL choleric toxin, and 100 U/mL penicillin and streptomycin (Invitrogen). All media were supplemented with 100 U/mL penicillin and streptomycin (Invitrogen). Cells were grown in a humidified incubator at 37°C with 5% CO_2_. Cell lines were purchased from the American Type Culture Collection (ATCC), and authenticity was documented by standard short tandem Repeat (STR) analysis. Cells were cultured in a humidified incubator at 37°C with 5% CO_2_ and tested periodically for *Mycoplasma* by 4,6-diamidino-2-phenylindole (DAPI) staining and PCR. Doxorubicin (Sigma) was dissolved in DMSO.

### 4.3 Plasmids

For p53 transient expression, pCDNA3-p53 was used (Mantovani et al., 2007). Retroviral plasmids used in this study were constructed using the pLPC-GFP vector (Girardini et al., 2011) containing a selectable puromycin cassette. The AKR1B1 coding sequence was amplified by PCR on cDNA from H1299 cells and cloned using EcoRI/XhoI sites. The following PCR primers were used: AKR1B1Eco_F: AAA​GAA​TTC​ATG​GCA​AGC​CGT​CTC​CTG​C, AKR1B1Xho_R: AAA​CTC​GAG​TCA​AAA​CTC​TTC​ATG​GAA​GGG. To generate pAKR1B1luc reporter, a fragment spanning from −1785 to +24 on the AKR1B1 promoter was amplified by PCR on genomic DNA from MDAMB231 cells and cloned into the pGL3-basic vector using MluI/XhoI sites. PCR primers used were AKR1B1luc_F: AAA​ACG​CGT​CAG​AGG​CAA​TGG​GGG​ATG​TTA, AKR1B1luc R:AAACTCGAGGGCGCGTACCTTTAAATAGCC. pAKR1B1luc was used as a PCR template to generate a series of 5′-terminally truncated AKR1B1 promoter fragments: pAKR1B1luc100, pAKR1B1luc70, and pAKR1B1luc40. Each fragment was ligated between SacI/HindIII sites into pGL3 basic vector to generate the corresponding reporter. PCR primers used to generate these constructs were AKR1B1luc100_F: AAA​GAG​CTC​CGC​AAC​CAA​TCA​GAA​GGC​TCC, AKR1B1luc70_F: AAA​GAG​CTC​CTC​CTT​CGC​GCA​GCG​GC, AKR1B1luc40_F: AAA​GAG​CTC​TTT​CTG​CCG​ACC​TCA​CGG, AKR1B1lucHind_R: AAA​AAG​CTT​GGC​GCG​TAC​CTT​TAA​ATA​GCC

### 4.4 Cell transfection and retroviral transduction

DNA and siRNA (10 pmol/cm^2^) transfection were performed with Lipofectamine 2000 (Invitrogen) according to the manufacturer’s instructions. The following siRNA sequences were used (Girardini et al., 2011): the sip53-targeting human *TP53* (GAC​UCC​AGU​GGU​AAU​CUA​C) and siControl-targeting LacZ gene from *Escherichia coli* (GUG​ACC​AGC​GAA​UAC​CUG​U). Stable genetic manipulation was performed by transduction with retrovirus-based plasmids as previously described (Girardini et al., 2011). In brief, retroviral particles were generated by co-transfection of HEK293-GP cells with the packaging plasmid pMD2ENV and specific constructs using the calcium phosphate method. Culture media containing retroviral particles were harvested 48 h after transfection, filtered, supplemented with 10% SFB and 4 μg/mL polybrene (Sigma), and used to transduce target cells. Upon transduction, cells were allowed to recover for 24 h in fresh media, and cells were selected with puromycin (Sigma).

### 4.5 Gene expression analysis

Total RNA was extracted using TRIzol reagent (Invitrogen) and subjected to DNase-I (Promega) treatment. RNA was retro-transcribed using M-MLV retrotranscriptase (Promega). Real-time PCR was performed using SYBR Green PCR master mix (Biodynamics) according to the following conditions: 2 min at 95°C for one cycle; 30 s at 95°C, 20 s at 60°C, and 30 s at 72°C for 40 cycles. The results were analyzed using the comparative ΔCt method. Values were normalized to *GAPDH* mRNA levels. The following real-time PCR primers were used: AKR1B1_F: TGA​GTG​CCA​CCC​ATA​TCT​CA, AKR1B1_R: TGT​CAC​AGA​CTT​GGG​GAT​CA, GAPDH_F: TCT​CTG​CTC​CTC​CTG​TTC, GAPDH_R: GCC​CAA​TAC​GAC​CAA​ATC​C

### 4.6 Western blot

Western blot was performed as previously described (Ibarra et al., 2017). The following primary antibodies were used: monoclonal anti-p53 (DO-1, Santa Cruz), monoclonal anti-AKR1B1 (CPTC-AKR1B1-3, DSHB), polyclonal anti-GFP (Ab290, Abcam), and polyclonal anti-β-actin (A2066, Sigma). HRP-conjugated anti-rabbit (111-035-003, Jackson) and HRP-conjugated anti-mouse (115-035-003, Jackson) secondary antibodies were used. Chemiluminescence was detected using Amersham ECL Prime Western Blotting Detection Reagent (GE Healthcare). For quantitative analysis, the intensity of Western blot bands was measured using ImageJ (Abràmoff et al., 2004) and normalized by the intensity of their respective β-actin bands.

### 4.7 Luciferase reporter assay

H1299 cells were co-transfected with the indicated plasmids and pCMV-β-galactosidase (Promega) as a control of transfection efficiency, using Lipofectamine 2000 (Invitrogen). Transfected cells were harvested in Passive Lysis Buffer 1X (Promega). Luciferase activity was measured using the Luciferase Assay Reagent (Promega) in a Multi-Mode Microplate Reader (Synergy 2, BioTek). The values were normalized relative to β-galactosidase activity.

### 4.8 ChIP assay

ChIP was performed as previously described (Borini Etichetti et al., 2019) on HEK-293 cells, HepG2 cells, H1299 cells transfected with pCDNA3-p53 or pCDNA3 as a control, or HCT116 cells treated with 0.5 µM doxorubicin or vehicle (DMSO) for 16 h. Chromatin was sonicated to 500–800 bp average fragment size and pre-cleared for 1 h at 4°C with protein A-Sepharose (GE Healthcare). Sepharose was removed by centrifugation, and an aliquot of the supernatant was saved as input. Chromatin was immunoprecipitated overnight at 4°C with anti-p53 antibody (DO1, Santa Cruz) or anti-GFP (sc-365549, Santa Cruz). Immunoprecipitated DNA was analyzed by real-time PCR. Promoter occupancy was calculated as percent of input chromatin using the ΔCt method. The following primers were used: AKR1B1ChIP_F: CGC​AAC​CAA​TCA​GAA​GGC​TCC, AKR1B1ChIP_R: GGC​GCG​TAC​CTT​TAA​ATA​GCC

### 4.9 *In silico* analysis of p53-binding sites

The presence of p53-binding sites on the AKR1B1 promoter was analyzed using JASPAR (Castro-Mondragon et al., 2022). For this analysis, the region spanning from −1785 to +24 on the AKR1B1 promoter was used.

### 4.10 Aldose reductase activity

Aldose reductase activity was determined using the method described by Hayman and Kinoshita (1965). In brief, cell extracts were prepared in KAB buffer (50 mM Hepes pH 7.5; 150 mM NaCl; 1 mM DTT; and 10 mM MgCl_2_), supplemented with protease inhibitor cocktail (Sigma) and 1mM PMSF. After sonication, centrifugation, and protein concentration determination, enzymatic activity was quantified in KAB buffer using 50 µg proteins from extracts, 175 µM NADPH, and 707 mM D + -xylose as substrate in 500 µL final volume. Initial enzymatic rates were followed at 37°C as the decrease in absorbance at 340 nm, corresponding to NADPH oxidation. KAB buffer supplemented with NADPH and xylose was used as blank.

### 4.11 Invasion assay

For *in vitro* invasion assays, Transwell chambers (8 µm pore size, Corning) were pre-coated with 1% Matrigel (Corning) in media without FBS for 1 h at 37°C. 4T1-AKR1B1 or 4T1-GFP cells were seeded on the upper side in media without FBS, and complete media were used in the lower chamber. After incubation for the indicated time, cells on the upper side of the Transwell were mechanically removed, and cells in the lower surface were fixed with 4% p-formaldehyde and stained with Hoechst. Invasive cells were quantified by fluorescence microscopy using ImageJ FIJI.

### 4.12 *In vivo* tumor formation

Procedures involving animals conformed to institutional guidelines that comply with international laws and policies (the Council for International Organization of Animal Sciences (CIOMS) and the International Council for Laboratory Animal Science (ICLAS)). All experimental protocols were approved by the Animal Ethics Committee at the School of Medicine, National University of Rosario (CICUAL-FCM-UNR. Res. 2534/2021). Six- to eight-week-old female BALB/c mice were obtained from the Center for Comparative Medicine (ICIVET), National University of Litoral. Animals were fed with commercial chow and water *ad libitum* and maintained in a 12-h light and 12-h dark schedule. Mice were randomly divided into two groups (n = 10 per group) and subcutaneously injected on the mammary fat pad with 1 × 10^6^ 4T1-AKR1B1 or 4T1-GFP cells in PBS. Palpable masses were measured twice a week using calipers, and tumor volume was quantified using formula V = (π.D.d^2^)/6, where D is the largest diameter, and d is the smallest diameter of the tumor. Three mice from the 4T1-AKR1B1 group died before the endpoint of the experiment, at days 6, 35, and 37 after injection. The first mouse was excluded from the experiment analysis, whereas the other two mice were included in the tumor growth calculation. However, autopsy and metastasis evaluation for these two mice could not be performed. After completion, mice were sacrificed, and organs were extracted for metastasis evaluation. To facilitate macroscopic visualization of lung metastases, the lungs were stained by intra-tracheal injection of India ink followed by wash as previously described (Miretti et al., 2008).

### 4.13 Proteomic analysis by liquid chromatography and mass spectrometry

Whole-cell extracts of 4T1-AKR1B1 and 4T1-GFP cells were prepared in biological triplicates. Cells were washed with PBS and resuspended in RIPA buffer. Upon lysis, the supernatants were recovered by centrifugation for further analysis. Tryptic peptides were obtained by in-gel digestion (Link and LaBaer, 2009). Peptide chromatography was performed on an Easy-nLC 1000 nanosystem (Thermo Scientific). Each sample was loaded into an Acclaim PepMap 100 precolumn (Thermo Scientific) and eluted into the analytical column (RSLC PepMap C18, 50 cm long, 75 µm inner diameter, and 2 µm particle size, Thermo Scientific). The mobile phase flow rate was 300 nL/min, using water + 0.1% formic acid (solvent A) and acetonitrile + 0.1% formic acid (solvent B). The gradient profile was set as follows: 5%–35% solvent B for 100 min, 35%–45% solvent B for 20 min, 45%–100% solvent B for 5 min, and 100% solvent B for 15 min. Mass spectrometry (MS) analysis was performed using a Q-Exactive mass spectrometer (Thermo Scientific). The full-scan method employed an m/z 300–1800 mass selection, an Orbitrap resolution of 70,000 (at m/z 200), a target automatic gain control (AGC) value of 3e6, and maximum injection times of 100 ms. After the survey scan, the 15 most intense precursor ions were selected for MS/MS fragmentation. Fragmentation was performed with a normalized collision energy of 27 eV, and MS/MS scans were acquired with a starting mass of m/z 200, AGC target of 2e5, resolution of 17,500 (at m/z 200), intensity threshold of 8e3, isolation window of 2.0 m/z units, and maximum IT of 100 ms. A dynamic exclusion time of 30 s was used to discriminate against previously selected ions. MS data were analyzed with Proteome Discoverer 2.4 using standardized workflows. Mass spectra *.raw files were searched against the database of *Mus musculus* from UniProt. Precursor and fragment mass tolerance were set to 10 ppm and 0.02 Da, respectively, allowing two missed cleavages, carbamidomethylation of cysteines as a fixed modification, methionine oxidation, and acetylation N-terminal as a variable modification. Data on identified and quantified proteins were analyzed with Perseus (Tyanova and Cox, 2018).

### 4.14 Functional enrichment analysis and interaction networks

The DEP list was defined as up- or downregulated proteins with an FC of 2 or higher, considering only proteins identified by two or more unique peptides (*p*-value ≤ 0.05) resulting from data processing by Proteome Discoverer. Functional enrichment analysis was performed using the KEGG Mapper tool (https://www.genome.jp/kegg/mapper/) (Kanehisa and Sato, 2020). A heatmap was made to visualize the DEPs in KEGG categories using the Matplotlib43 and Seaborn44 packages. All packages were run on Python 3.10. Interaction networks were obtained from STRING (Szklarczyk et al., 2021) with a confidence cutoff of 0.6 and no extra interactors and loaded onto Cytoscape v3.9.1 (Shannon et al., 2003), with a confidence cutoff of 0.6. Network clustering was performed using the MCL algorithm set to a granularity parameter of 2.5. For network analysis, the DEP list was extended considering proteins with log_2_FC ≥ 0.85 or log_2_FC ≤ −0.85 (*p*-value ≤ 0.05), in order to increase the number of interactors. Proteins without interaction partners within the network (singletons) were omitted from the visualization.

### 4.15 Determination of ROS

For ROS determination, a modification of the method reported by Wang and Joseph (1999) was used. Cells were incubated with 5 µM 2′,7′-dichlorofluorescein diacetate (Sigma) in 1% FBS media for 45 min at 37°C in the dark. After washing with PBS, fluorescence was measured with an excitation wavelength of 488 nm and emission wavelength of 525 nm using a Multi-Mode Microplate Reader (Synergy 2, BioTek). Non-stained cell suspensions were used as blank.

### 4.16 Statistical analysis

All experiments were conducted in triplicates unless otherwise indicated. The results were plotted as mean values +/- standard error of the mean using GraphPad Prism7 and statistically analyzed using a two-tailed *t*-test or ANOVA followed by a multiple-comparison post-test, as appropriate. *p*-values less than 0.05 were considered statistically significant.

## Data Availability

The mass spectrometry proteomics data presented in this study are deposited in the ProteomeXchange Consortium (https://www.proteomexchange.org/) via the PRIDE partner repository, with the dataset identifier PXD045048.
