# Supplementary figures and images for: The p53 tumor suppressor regulates AKR1B1 expression, a metastasis-promoting gene in breast cancer
Source: Front Mol Biosci. 2023 Sep 14;10:1145279. doi: 10.3389/fmolb.2023.1145279 (PMC10538543; doi:10.3389/fmolb.2023.1145279)

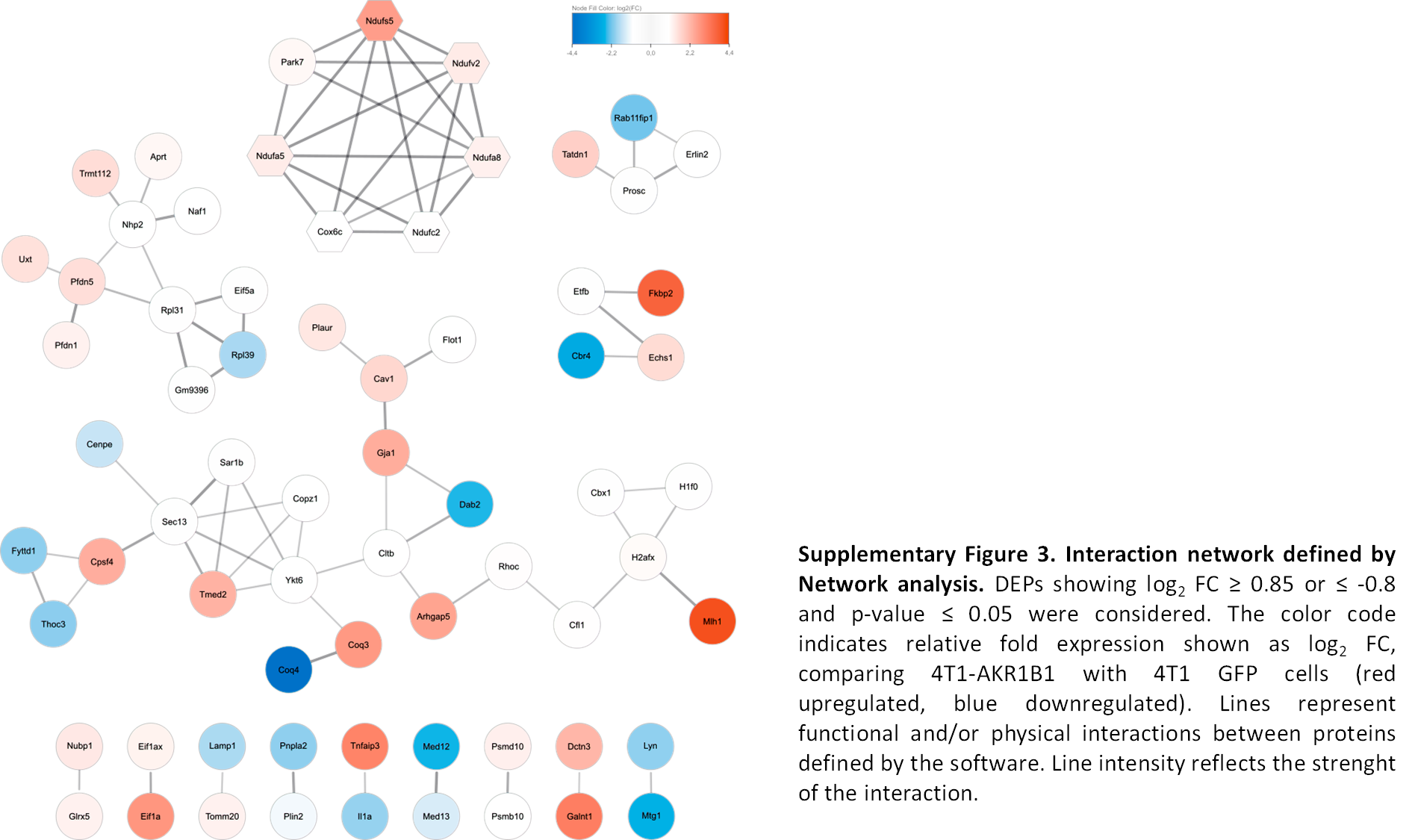

Supplement: Supplementary file 1 [file Image3.TIF]

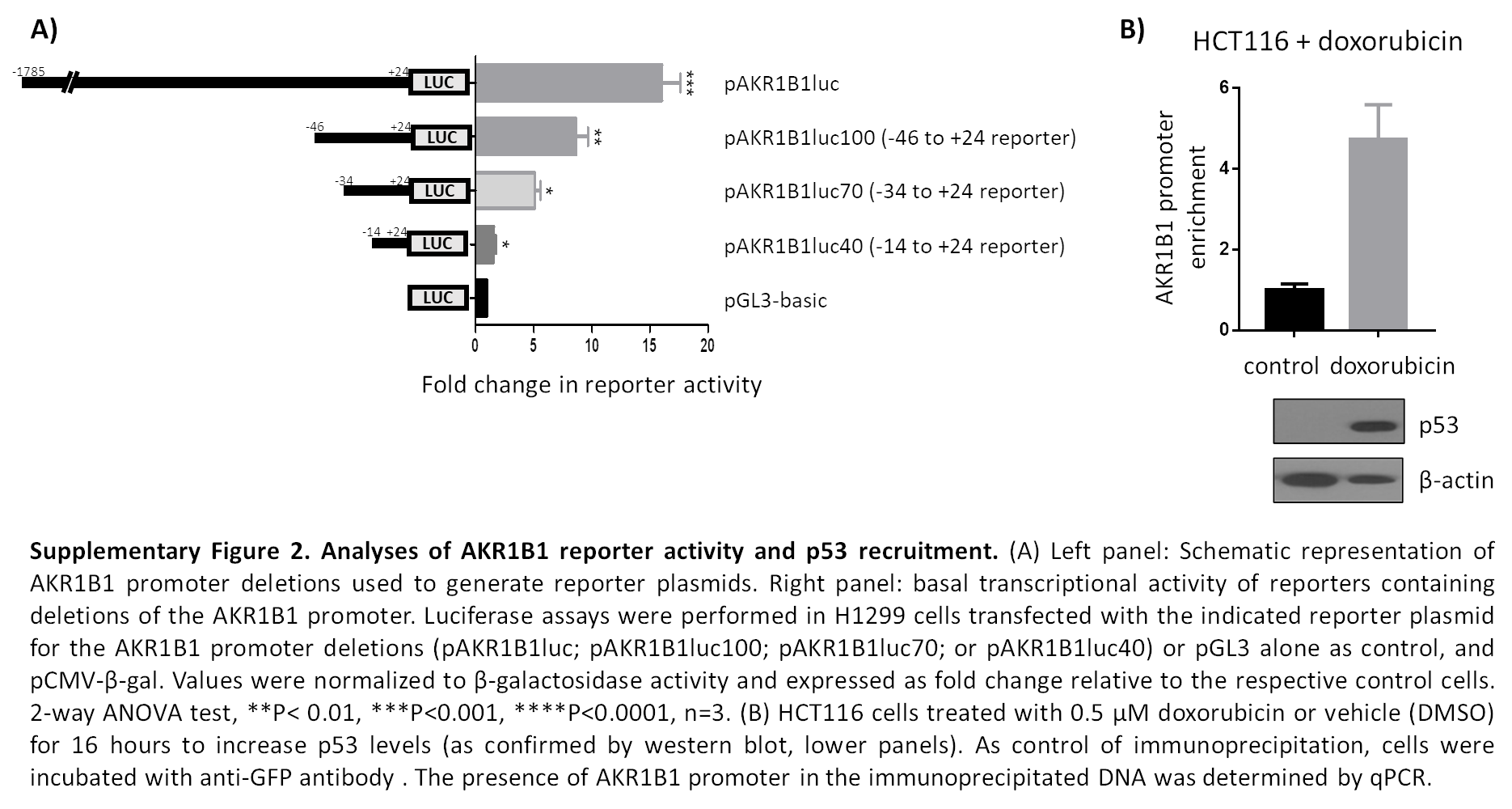

Supplement: Supplementary file 2 [file Image2.TIF]

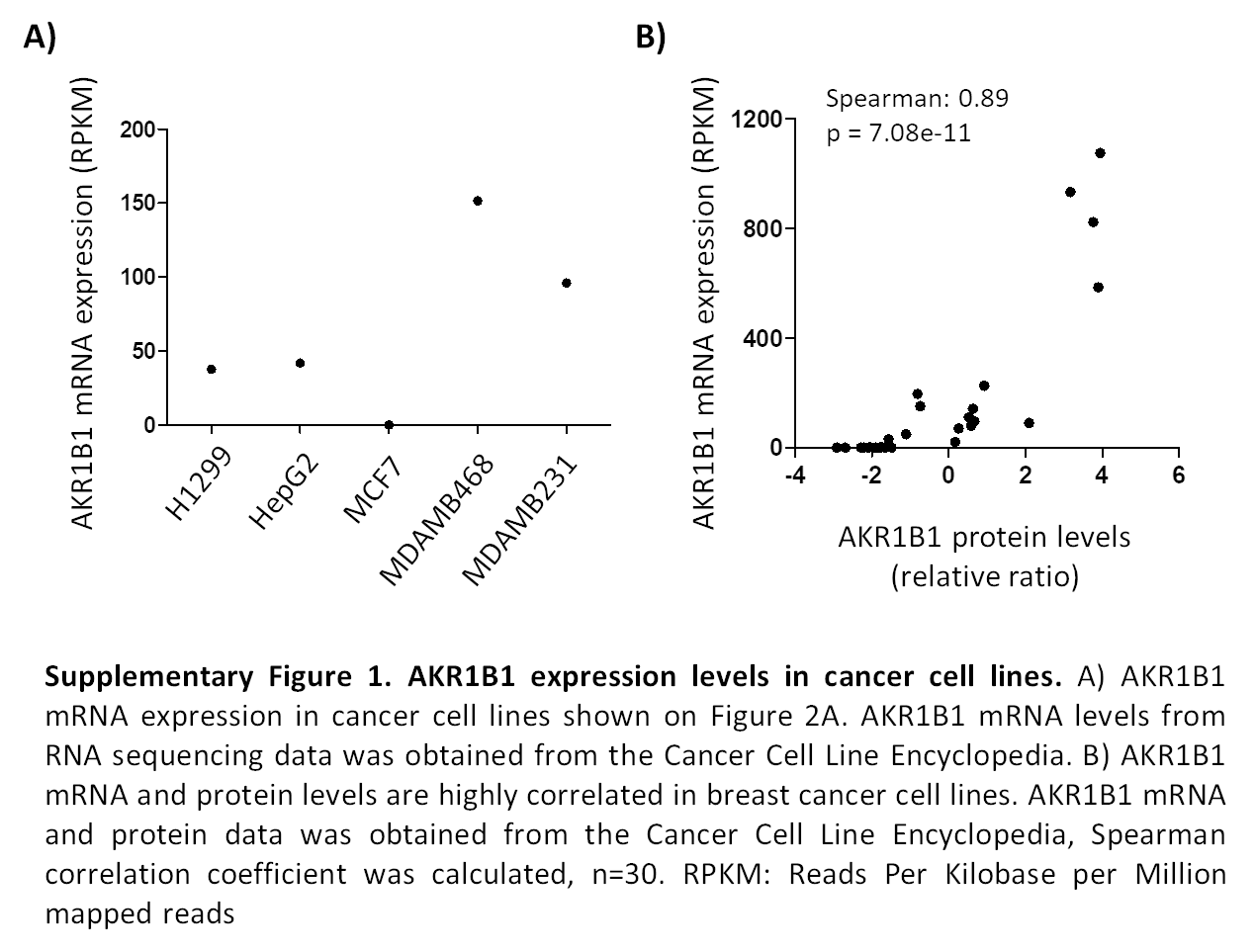

Supplement: Supplementary file 3 [file Image1.TIF]
